# Progress and challenges of sequencing and analyzing circulating tumor cells

**DOI:** 10.1007/s10565-017-9418-5

**Published:** 2017-11-22

**Authors:** Zhongyi Zhu, Si Qiu, Kang Shao, Yong Hou

**Affiliations:** 1 BGI-Shenzhen, Shenzhen, 518083 China; 2 China National GeneBank, BGI-Shenzhen, Shenzhen, 518120 China

**Keywords:** Circulating tumor cell, Next-generation sequencing, Single-cell sequencing

## Abstract

Circulating tumor cells (CTCs) slough off primary tumor tissues and are swept away by the circulatory system. These CTCs can remain in circulation or colonize new sites, forming metastatic clones in distant organs. Recently, CTC analyses have been successfully used as effective clinical tools to monitor tumor progression and prognosis. With advances in next-generation sequencing (NGS) and single-cell sequencing (SCS) technologies, scientists can obtain the complete genome of a CTC and compare it with corresponding primary and metastatic tumors. CTC sequencing has been successfully applied to monitor genomic variations in metastatic and recurrent tumors, infer tumor evolution during treatment, and examine gene expression as well as the mechanism of the epithelial-mesenchymal transition. However, compared with cancer biopsy sequencing and circulating tumor DNA sequencing, the sequencing of CTC genomes and transcriptomes is more complex and technically difficult. Challenges include enriching pure tumor cells from a background of white blood cells, isolating and collecting cells without damaging or losing DNA and RNA, obtaining unbiased and even whole-genome and transcriptome amplification material, and accurately analyzing CTC sequencing data. Here, we review and summarize recent studies using NGS on CTCs. We mainly focus on CTC genome and transcriptome sequencing and the biological and potential clinical applications of these methodologies. Finally, we discuss challenges and future perspectives of CTC sequencing.

## Introduction

Circulating tumor cell (CTC) studies began in 1869 (Ashworth 1869) and now are rapidly increasing, with a steadily expanding list of CTC-related studies retrievable from NCBI PubMed. An average of over 1000 CTC-related papers were published per year in the last 5 years. During cancer progression, cancer cells in the primary tumor may invade into nearby blood vessels via the epithelial to mesenchymal transition (EMT) process and then circulate throughout the entire blood system. After traveling some distance, CTCs may leave the blood vessels via the mesenchymal to epithelial transition (MET) process, the reverse process of EMT (Nieto 2013), which helps cancer cells seed in different tissues and generate metastatic lesions. In particular, two recent studies reported evidence that EMT is involved in the metastatic potential of CTCs by detecting EMT markers in human CTCs (Armstrong et al. 2011; Kallergi et al. 2011).

Previous CTC studies have mainly focused on the development of CTC enrichment technology (Hong and Zu 2013; Alix-Panabieres and Pantel 2014; Krebs et al. 2014; van der Toom et al. 2016), the correlation between CTC number and clinical prognosis (Arya et al. 2013; Harouaka et al. 2014; Alix-Panabieres and Pantel 2016), and biological processes driving cancer metastasis and recurrence (Hodgkinson et al. 2014; Massague and Obenauf 2016). For example, scientists have discovered a correlation between CTC number and the prognosis of breast cancer (Eroglu et al. 2013; Bidard et al. 2016), colon cancer (Hardingham et al. 2015), and prostate cancer (Hu et al. 2013), whereby the CTC number can be used to assess cancer prognosis. Based on these principles, the US Food and Drug Administration (FDA) approved the CellSearch™ system as an aid for monitoring breast cancer (Cristofanilli NEJM 2004), prostate cancer (Cohen JCO 2008), and colon cancer (Scher Lancet Oncol 2009) progression.

However, until recently, few CTC sequencing studies have been published. We searched the CTC studies based on next-generation sequencing (NGS) technology in NCBI PUBMED and found 19 CTC publications (Table 1). Among these publications, over half of the CTC DNA sequencing studies focused on capturing genes with potential as targeted cancer therapies or those involved in metastasis or recurrence, and a limited number of studies used whole-genome sequencing (WGS) or whole-exome sequencing (WES). Additionally, several studies have highlighted CTC transcriptome sequencing as a promising approach for investigating metastasis mechanisms such as EMT. In this review, we summarized and described the methodologies and pipeline of NGS of CTCs, including cell enrichment, isolation and capturing methods, and whole-genome or transcriptome amplification methods. Furthermore, we focused on the biological insights achieved from CTC sequencing as well as its potential clinical applications. Finally, we highlighted major challenges for CTC sequencing and bioinformatics analysis and discussed the future perspectives for CTC sequencing in the NGS area.

**Table 1:** Overview of next-generation sequencing CTC studies

Sequencing strategies	Cancer type	Ref
Target (68 genes)	Stage IV colorectal carcinoma	(Heitzer et al. 2013)
WES	Prostate cancer	(Zhao et al. 2013)
WGS/WES	Lung cancer	(Ni et al. 2013)
WES	Prostate cancer	(Lohr et al. 2014)
WGS	Prostate cancer	(Dago et al. 2014)
Target (46 genes)	Hepatocellular carcinoma	(Kelley et al. 2015)
WGS/target (20 genes)	MelanomaSmall-cell lung cancer	(Rothwell et al. 2016)
WGS/WES	Prostate cancer	(Jiang et al. 2015)
Target (50 genes)	Metastatic breast cancer	(De Luca et al. 2016)
Target (50 genes)	Metastatic breast cancer	(Shaw et al. 2017)
Target (50 genes)	Melanoma	(Palmirotta et al. 2017)
Target (6 genes)	Liver, colorectal, lungGastric, breast, prostate cancer	(Wong et al. 2017)
WGS/WES	Breast, gastric, prostate, colon cancer	(Gao et al. 2017)
SC RNA-Seq	Pancreatic cancer	(Yu et al. 2012)
SC RNA-Seq	LNCaP,Prostate cancer	(Cann et al. 2012)
SC RNA-Seq	Breast cancer	(Yu et al. 2013)
SC RNA-Seq	KPC mice, pancreaticBreast, prostate cancer	(Ting et al. 2014)
SC RNA-Seq	Prostate cancer	(Miyamoto et al. 2015)
SC RNA-Seq	Colorectal cancer cell line	(Grillet et al. 2016)

## Methodology of CTC genome and transcriptome sequencing

**Fig. 1:**
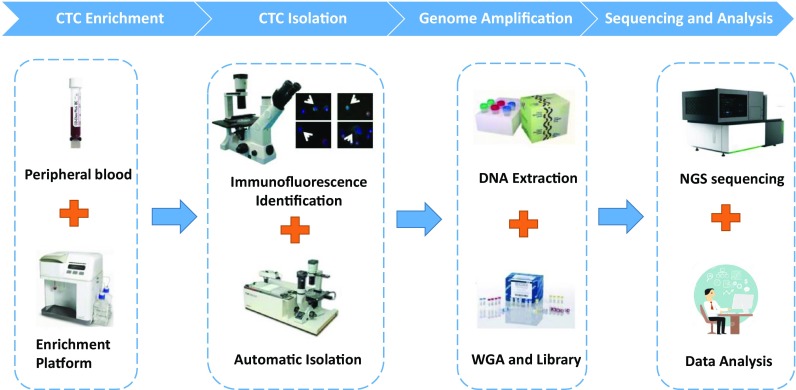
The workflow of circulating tumor cell sequencing

Generally, the CTC sequencing workflow can be separated into four steps: CTC enrichment, CTC isolation (particularly single-cell CTC or pure CTC isolation), genome or transcriptome amplification, and sequencing and analysis (Fig. 1). Several successful CTC enrichment methods have been reported for enriching CTCs from cancer patient blood. Generally, two strategies are used for these methods. The most common method is to utilize cell surface CTC markers for enrichment (EPCAM^+^, CK^+^, CD44^+^) or to delete immune cells (CD45^−^) (Alix-Panabieres and Pantel 2014; Krebs et al. 2014; Ferreira et al. 2016), and representative platforms include CellSearch (Riethdorf et al. 2007), MagSweeper (Talasaz et al. 2009; Deng et al. 2014), and GILUPI cell collector (Saucedo-Zeni et al. 2012), among others. The other enrichment strategy uses the physical characteristics of CTCs (size, density, acoustics, fluid force) to separate CTCs from the leukocyte background (Harouaka et al. 2013; Krebs et al. 2014), and representative platforms include ClearCell (Hou et al. 2013; Khoo et al. 2015), ISET (Vona et al. 2004; Chinen et al. 2013). Recently, microfluidic channels and waves integrated with cell surface markers or physical characteristics, such as IsoFlux (Harb et al. 2013), are widely used in CTC enrichment systems and significantly improve the efficiency and accuracy of CTC identification (Li et al. 2015; Shields et al. 2015). In addition, systems such as CTC-iChip (Ozkumur et al. 2013; Karabacak et al. 2014) that combine cell surface markers and physical characteristics exhibit promising performance, recovering more CTCs with less DNA or RNA damage. Most of these methods require 7.5 ml (or more) of peripheral blood for effective enrichment, whereas the GILUPI cell collector overcomes the barrier of small blood samples by collecting CTCs in vivo from the peripheral blood stream.

After CTC enrichment from the blood, zero to several hundred CTCs may be retained in a thousand to ten thousand background cells, resulting in a low efficiency for sequencing and analysis of these cell pools (Alix-Panabieres and Pantel 2014). Therefore, scientists typically use the tools commonly used for SCS, such as laser capture microdissection (LCM) and flow cytometry analysis (FACS), to further isolate CTCs from background cell pools. As mentioned in previous SCS reviews, different methods have distinct pros and cons (Macaulay and Voet 2014; Wang and Navin 2015; Chen et al. 2016; Gawad et al. 2016). Compared with LCM, FACS automatically isolates specific individual cells with correct markers into tubes or wells in a high-throughput manner, whereas an LCM approach allows the observation of cellular morphology and physiology to prevent possible contamination or cell damage, although this approach is time-consuming and labor intensive. Recently, an increasing number of automated systems based on the microfluidics approach have been developed to enrich and isolate CTCs. For example, the DEPArray system from Silicon Biosystems utilizes force from non-uniform electric fields to drive, capture, and recover cells, avoiding physical contact of the cells with other substrates. Thus, cells isolated from this system are biologically healthy and retain complete genetic information, which is used for sequencing and gene expression profiling (Gascoyne et al. 2009; Carpenter et al. 2014). In addition, scientists built in-house pipelines or systems to isolate CTCs for their specific study design. In Lohr’s work, the enriched CTCs were separated into micro-well chips, and subsequently specific CTCs enriched with fluorescence markers were selected for further sequencing and analysis (Lohr et al. 2014). Other studies using a combination of different enrichment methods including (Zhao et al. 2013) marker-free microfluidic isolation, direct sequencing (Palmirotta et al. 2017), and automatic CTC counting (Wong et al. 2017) also demonstrated that the development of CTC enrichment and isolation technology significantly improved the efficacy and efficiency of CTC sequencing studies.

After obtaining target cells from whole blood, the genetic material should be amplified to generate enough template to create an NGS library. Methods for amplifying the whole genome or transcriptome have been summarized in previous SCS reviews (Macaulay and Voet 2014; Wang and Navin 2015; Gawad et al. 2016). Briefly, for whole-genome amplification (WGA), scientists use linear or PCR-based amplification methods such as MDA (Spits et al. 2006), MALBAC (Lu et al. 2012), and DOP-PCR (Cheung and Nelson 1996), which are commonly used in SCS studies (Hou et al. 2015; Huang et al. 2015). For whole-transcriptome amplification (WTA), methods such as CEL-seq (Hashimshony et al. 2012), STRT-seq (Islam et al. 2014), and SMART-seq (Picelli et al. 2013) were developed to amplify either full-length transcripts or their 3′ region. Most of these amplification methods have been commercialized as kits such as the GenomePlex Single Cell WGA Kit, the Qiagen REPLI-g Single Cell Kit, and the SMARTer Ultra Low Input RNA Kit. After obtaining enough genetic material, the NGS library is prepared and sequenced with standardized protocols. After obtaining sequencing data from CTCs, the most important procedure is to evaluate bias during sample preparation and design an appropriate statistical model to handle these biases (described in the challenges section).

According to previous publications (Ni et al. 2013; Ting et al. 2014; Miyamoto et al. 2015) and our experience, the successful rate of overall amplification and library preparation is under 60% because of multiple sample handling and staining processes. To improve the success rate, some scientists pool isolated CTCs together or directly pool all of the recovered cells (including white blood cells (WBCs)) (Palmirotta et al. 2017; Shaw et al. 2017; Wong et al. 2017). The advantage of this strategy is that it has a high library preparation success rate, although it introduces increased noise into subsequent bioinformatics analyses. Other groups demonstrated that only amplifying specific regions of the genome with multiplex PCR reduces the complexity of the experiment and increases the success rate (Palmirotta et al. 2017).

## Monitoring clinically relevant genetic variations during cancer progression

Cancer metastasis and recurrence are major challenges to clinical treatment and the major causes of death in cancer patients. Compared with the primary tumor, genome sequencing studies have shown that cancer cells from metastatic and recurrent tumors acquire novel somatic variations that enhance cell progression during treatment (Mwenifumbo and Marra 2013). In clinical practice, it is usually difficult to acquire a re-biopsy from metastatic or recurrent tumors, leading to ambiguous diagnostic results during treatment. Liquid biopsy recently emerges as a significant breakthrough in cancer translational research. By sequencing the circulating tumor DNA (ctDNA) from the CTCs, researchers observed the somatic variation in the landscapes of the tumor without biopsy sequencing (Crowley et al. 2013). Scientists have demonstrated that somatic variations detected in ctDNA or CTCs highly correlate with primary, metastatic, or recurrent tumors and could be used for clinical diagnosis and disease monitoring (Alix-Panabieres and Pantel 2016). Although ctDNA is much easier to obtain, CTCs contain complete genetic information, including the genome, transcriptome, and even epigenome of the circulating cells, which provides more comprehensive genetic information for scientific investigations.

**Fig. 2:**
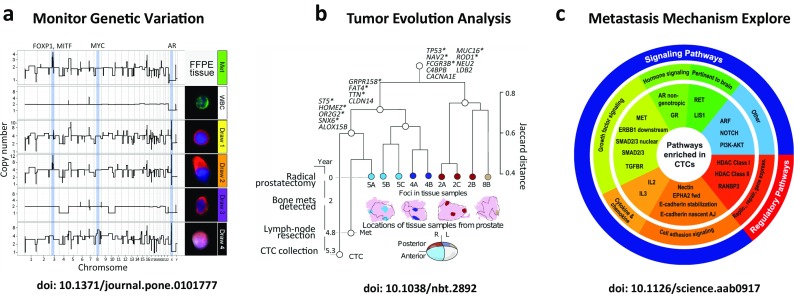
Biological and potential clinical applications of CTC sequencing. **a** The CNV pattern of CTCs in a prostate cancer patient changed under therapeutic pressure. **b** CTC sequencing served as an efficient tool to uncover biological insights concerning tumor evolution. **c** RNA sequencing of CTCs enabled the exploration of metastasis-related pathways

Previous CTC studies have highlighted the biological involvement of CTCs in cancer metastasis and recurrence (Hou et al. 2011; Franken et al. 2012; Massague and Obenauf 2016). As liquid biopsy tools, CTC sequencing could serve as an efficient and unique tool to monitor cancer progression and to discover somatic mutations with possible clinical relevance that occurred or disappeared pre- and post-treatment (Fig. 2a). FD Luca et al. reported that most of the CTC baseline mutations were eliminated, and novel mutations emerged after chemotherapy treatment in one metastatic breast cancer patient (De Luca et al. 2016). However, a common mutation, p.V777L in the *ERBB2* gene, was detected in all of the post-therapy CTCs, indicating that the clone bearing this mutation might have played a role in the resistance to the administered therapy. Xiaohui Ni et al. reported the clinical relevance of *PIK3CA*, *RB1*, and *TP53* mutations in lung cancer treatment, such as erlotinib drug resistance and two vivid examples of the potential clinical uses for CTC sequencing during disease monitoring (Ni et al. 2013). After CTC sequencing, these authors identified a patient carrying the *PIK3CA* mutation, which has been associated with drug resistance to erlotinib and chemotherapy strategy selection. In addition, Miyamoto et al. analyzed the RNA-Seq profiles of 77 intact CTCs isolated from 13 prostate cancer patients and showed that single CTCs exhibit high heterogeneity for AR gene mutations and splicing variants, demonstrating that signaling pathway heterogeneity might be responsible for treatment failure (Miyamoto et al. 2015).

## Inferring tumor heterogeneity and evolution dynamically

Typically, cancer is considered the result of Darwinian evolution, as cancer continually acquires new somatic mutations in single cells, followed by selection, which enhances the fitness and growth advantage of a specific group of malignant cells (Stratton et al. 2009). Understanding intra-tumoral heterogeneity (ITH) and evolution is essential for the early detection of disease recurrence and the efficient treatment of cancer (Lipinski et al. 2016). There are three major hypothetical models explaining ITH, including clonal evolution, the cancer stem cell, and mutator phenotype models (Russnes et al. 2011). Recently, the development of deep sequencing and SCS make it possible to investigate phylogenetic evolution during cancer progression (McGranahan and Swanton 2017) (Fig. 2b). For example, Navin et al. decoded the evolutionary pattern of breast cancer and related metastatic lesions using single-cell genome sequencing (Navin et al. 2011; Wang et al. 2014; Gao et al. 2016). Hou et al. applied single-cell exome sequencing to blood neoplasm and kidney cancer (Hou et al. 2012; Xu et al. 2012). Macro et al. used multiregion deep sequencing to examine the evolutionary pattern of kidney tumors (Gerlinger et al. 2012). However, due to a large amount of heterogeneity in solid tumors and the difficulty of re-biopsy, the evolution of cancer metastasis and recurrence remains elusive. Compared to only sequencing primary and metastasis tumor cells, sequencing CTCs provides additional data to further explore ITH complexity. In addition, because CTC survival in peripheral blood is an essential step for tumor metastasis, sequencing CTC genomes can delineate a more detailed tumor evolution process, facilitating the understanding of the tumor metastasis mechanism.

Many CTC sequencing studies have highlighted the genetic heterogeneity of CTCs, which further increases the complexity of CTC research in cancer biology. For example, Luca et al. sequenced a 50-gene NGS panel on CTCs isolated from four metastatic breast cancer patients and reported a 50% (20 of 50 genes) CTC variation detection rate in a mean depth of 1500X of 14 CTCs. These authors concluded that there exists a high inter- and intra-patient heterogeneity in CTC mutational status (De Luca et al. 2016). Constructing the tumor evolution process through the detected CTC somatic nucleotide variants (SNVs) and mapping the early trunk mutations (mutation present early in tumor evolution) have considerable clinical utility. For example, Lohr et al. reported that CTCs shared 90% primary trunk mutations and 70% metastasis trunk mutations in a prostate cancer patient, respectively. However, due to a limited captured number of CTCs in most cancer types, it is difficult to analyze the SNV evolutionary structure in individual patients.

Moreover, copy number variation (CNV) is also frequently altered during cancer evolution. Ni et al. surveyed the CTC CNVs from a small-cell lung cancer (SCLC) patient during sequential chemotherapy treatment and observed that the evolution of CNV was consistent along the therapeutic stage, indicating that the reproducible CNV pattern was not affected by drug treatment (Ni et al. 2013). Dago and colleagues collected blood samples at multiple time points during the treatment of metastatic prostate cancer patients and found that the initial CNV evolution in CTCs changed along with a clinical response feature (decreased pain and PSA level), and CNV evolution changed again with a clinical observation of disease progress (increasing pain and PSA levels). Thus, these authors concluded that the CNV evolution of CTC would be affected by therapeutic pressure in prostate cancer (Dago et al. 2014). Gao et al. analyzed the CNV of CTCs across 23 patients and concluded that the CNV tumor evolution process follows a convergent evolution model through primary tumor to CTCs but does not follow the classical gradual acquisition mode or the recent alert punctuated model (Gao et al. 2017). However, more patients showed different variation spectrums during evolution, most of which still cannot be comprehensively explained. Further investigations into the biological impact of these complex somatic mutations during cancer treatment will significantly contribute to our understanding of CTC biology in cancer progression and future clinical application in areas of disease monitoring.

## Understanding altered molecular pathways during tumor progression

Previous studies have highlighted particular molecular pathways involved with cancer metastasis, such as TGF-β signaling (Akhurst and Derynck 2001), Wnt signaling (Polakis 2012), and EMT (Gonzalez and Medici 2014). However, most of these studies were based on mouse models or specific biomarkers (Thiery et al. 2009; Li et al. 2010; Harper et al. 2016). Analyzing CTC gene expression provides a unique window into understand the molecular pathways altered during metastasis despite the existence of significant ITH. The emergence of single-cell RNA sequencing (scRNA-seq) technology also enabled the acquisition of comprehensive gene expression and splicing information using a limited number of isolated CTCs (Ramskold et al. 2012). Thus, CTC transcriptome sequencing provides a unique window to digitize molecular pathways during cancer progression (Fig. 2c).

Yu et al. first utilized single-molecule RNA sequencing and a mouse pancreatic cancer model and found that the *WNT2* gene mediated the metastasis-associated survival signal, consistent with observations of the upregulation of multiple Wnt genes in pancreatic patients (Yu et al. 2012). This report is the first comprehensive study utilizing CTC RNA-Seq to uncover complete molecular pathways altered during cancer metastasis. This study was further expanded by analyzing the scRNA-seq of CTCs and comparing the results with matched primary tumors in a pancreatic cancer model (Ting et al. 2014). The authors observed that extracellular matrix genes are highly expressed in mouse and human CTCs and SPARC (an extracellular matrix protein), which may contribute to pancreatic tumor metastasis. In addition, Miyamoto et al. presented another study of 13 drug-resistant prostate cancer patients utilizing the scRNA-seq of CTCs and found that drug resistance in prostate cancer was triggered by the activation of the non-canonical Wnt signaling pathway (Miyamoto et al. 2015).

The EMT of adherent epithelial cells to a migratory mesenchymal state has been implicated in tumor metastasis in pre-clinical models. Yu et al. characterized the dynamic cell fates in breast cancer CTCs and found an association of mesenchymal CTCs with disease progression (Yu et al. 2013). By directly sequencing the RNA of CTC-enriched cell populations from a metastatic breast cancer patient at five serial time points and comparing the samples to ten healthy donors, these authors identified 170 CTC transcripts at a mesenchymal-predominant time point, which showed dramatic enrichment for EMT-related expression changes and extracellular matrix (ECM) and ECM-related membrane receptors. Another interesting observation in this study is that both single CTCs and multicellular cluster CTCs express known EMT regulators, including TGF-β pathway components. These authors further showed that CTC clusters originated monoclonally from the primary tumor and showed markedly increased metastatic capability compared with single CTCs, which had a poor prognosis in a mouse model (Aceto et al. 2014). Combined with the scRNA-seq of CTC clusters and single CTCs, these authors also demonstrated that plakoglobin was implicated in cluster formation during breast cancer metastasis. This study not only demonstrated molecular signaling in CTCs but also provided a putative novel drug target to control breast cancer metastasis. Another study from Grillet et al. established three CTC lines from three chemotherapy-naïve advance metastatic colorectal cancer patients and demonstrated the enrichment of drug metabolism pathways, which corresponds to cytotoxic compound resistance using RNA-Seq of CTCs and primary tumors (Grillet et al. 2016). Taken together, these findings suggest potential markers of tumor progression and treatment response and indicate the great potential of taking these observations to the clinic.

## Challenges and future perspectives of CTC sequencing

As shown in this review, CTC sequencing can now be used as an efficient liquid biopsy tool to investigate the spectrum of somatic variation and gene expression changes in primary, metastatic, and recurrent cancer patient tumors non-invasively. The somatic alterations could either be used to understand the origin, ITH, and evolution of tumors or to monitor disease progression during cancer treatment. The most important clinical implication of CTC sequencing is for personalized medicine, or so-called precision medicine, according to the variation spectrum detected, which indicates the selection of target therapy based on the CTC variation spectrum. With the development of CTC capture and SCS technologies, we can expect the establishment of more comprehensive cancer origins and evolution models in the near future. Moreover, more novel biomarkers or potential drug targets for cancer metastasis or drug resistance prevention or treatment can be identified through CTC sequencing.

However, as mentioned above, sequencing a CTC genome or transcriptome faces technical challenges. Obtaining enough cells for library preparation and sequencing is the first critical step in CTC sequencing. However, various conclusions can be drawn from different studies based on different isolation method and cancer types. Some cancer types tend to generate more CTCs than other cancer types (Allard et al. 2004), and the clinical stage is also associated with the CTC number collected from patients. Although there are several controversial conclusions, it is common that patients in late stages of cancer or with metastatic lesions contain more CTCs (Pantel et al. 2009). The number of CTCs typically varies from zero to several hundred (even thousand in some cases) per 7.5 ml of blood. In general, obtaining enough CTCs for sequencing remains a significant problem for most cancer types, which limits the number of CTC sequencing studies. In addition, cell loss or genetic material damage during CTC enrichment, isolation, genome, or transcriptome amplification has consistently been reported in studies based on various CTC enrichment systems. Further, leukocyte contamination, lack of specific biomarkers, low-throughput and time-consuming manual capture operation protocol also hinder the progress of CTC sequencing studies (Cann et al. 2012). Obtaining high-quality sequencing libraries is another critical step in CTC sequencing. Based on the description of Miyamoto et al. (Miyamoto et al. 2015), 77 out of 221 cells (35%) were defined as qualified cells for bioinformatics analysis. Many other studies, including Lohr et al. or Dago et al. (Dago et al. 2014; Lohr et al. 2014) and our own experience, have also shown the same success rate for CTC sequencing.

**Fig. 3:**
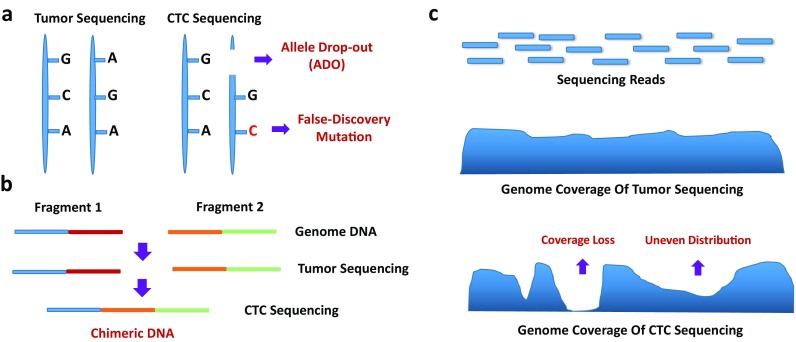
CTC sequencing biases introduced during amplification and library preparation. **a** Allele drop out (ADO) during genome amplification leads to the loss of the detection of somatic mutant alleles in CTCs, and false-positive results can also be introduced into both WGA and library processes. **b** Chimeras will lead to artificial CNV and SV detection in CTC sequencing. **c** The limitations of the WGA method may lead to low genome coverage or uneven read distribution effects when compared to bulk tumor sequencing

More importantly, bioinformatics analyses of CTC sequencing data require additional quality evaluation and assessment, particularly the biases introduced during sample and sequencing library preparation (Fig. 3). Allele drop out (ADO) during genome amplification may prevent the detection of the somatic mutant alleles of CTCs, which may contribute to cancer progression or drug resistance (Hou et al. 2012; Nawy 2014). In addition, the limitation of the WGA method may lead to low genome coverage (Kelley et al. 2015), high false-positive rates, and low sensitivity of mutation detection (Lohr et al. 2014; Shaw et al. 2017). Uneven reads distribution and chimeras from WGA may also lead to artifacts in the CNV and SV detection of CTCs (Ni et al. 2013; Lohr et al. 2014; Jiang et al. 2015). However, as reviewed in SCS studies, scientists can design specific statistical models, such as kindred replication (Chen et al. 2017; Dong et al. 2017), the Bayesian model (Kharchenko et al. 2014), and the binomial model (Vu et al. 2016), to reduce these biases and more accurately detect SNVs, CNVs, and SVs.

Despite the development of advanced microfluidics approaches, several novel sequencing technologies show promise for solving the technological hurdles to CTC sequencing. For example, scientists can now perform in situ DNA or RNA sequencing, even on samples fixed on slides (Lee et al. 2015). This approach decreases the complexity of sample handling processes and the cell damage or loss that can occur during CTC enrichment and isolation. In addition, emerging single-molecule sequencing technologies show promise for analyzing DNA or RNA molecules without amplification. Thus, biases or artifacts, such as ADO, false-positive mutations, and uneven amplification, may be significantly reduced during CTC sequencing. Furthermore, single-molecule sequencing (Liu and Wu 2011; Gawad et al. 2016; Heather and Chain 2016) may expand the CTC sequencing approach to analyze wide epigenome information, such as methylation and chromatin occupation.
